# Coevolution of Male and Female Genital Morphology in Waterfowl

**DOI:** 10.1371/journal.pone.0000418

**Published:** 2007-05-02

**Authors:** Patricia L.R. Brennan, Richard O. Prum, Kevin G. McCracken, Michael D. Sorenson, Robert E. Wilson, Tim R. Birkhead

**Affiliations:** 1 Department of Ecology and Evolutionary Biology, and Peabody Natural History Museum, Yale University, New Haven, Connecticut, United States of America; 2 Department of Animal and Plant Sciences, University of Sheffield, Western Bank, Sheffield, United Kingdom; 3 Institute of Arctic Biology, Department of Biology and Wildlife, and University of Alaska Museum, University of Alaska Fairbanks, Fairbanks, Alaska, United States of America; 4 Department of Biology, Boston University, Boston, Massachusetts, United States of America; University of Oxford, United Kingdom

## Abstract

Most birds have simple genitalia; males lack external genitalia and females have simple vaginas. However, male waterfowl have a phallus whose length (1.5–>40 cm) and morphological elaborations vary among species and are positively correlated with the frequency of forced extra-pair copulations among waterfowl species. Here we report morphological complexity in female genital morphology in waterfowl and describe variation vaginal morphology that is unprecedented in birds. This variation comprises two anatomical novelties: (i) dead end sacs, and (ii) clockwise coils. These vaginal structures appear to function to exclude the intromission of the counter-clockwise spiralling male phallus without female cooperation. A phylogenetically controlled comparative analysis of 16 waterfowl species shows that the degree of vaginal elaboration is positively correlated with phallus length, demonstrating that female morphological complexity has co-evolved with male phallus length. Intersexual selection is most likely responsible for the observed coevolution, although identifying the specific mechanism is difficult. Our results suggest that females have evolved a cryptic anatomical mechanism of choice in response to forced extra-pair copulations.

## Introduction

Complex genitalia can result from different evolutionary mechanisms [reviewed in 1], [2], although in recent years sexual selection is increasingly regarded as the primary force behind the evolution of genital diversity [1]–[4]. Elaborate genitalia have been hypothesized to evolve through post-copulatory competition among males for fertilization of female ova [1]; female choice for males that are either good stimulators or of higher quality [1], [5]; or from an arms race between the sexes over the control of insemination and fertilization [1], [2], [6]–[8].

Genital morphologies that give a sexual advantage to one sex at the expense of the other could lead to coevolution between the sexes and an evolutionary arms race in copulation behaviour, morphology, or physiology [9]–[11]. If males have genital traits that allow them to manipulate females and bias paternity, then coevolved modifications in female genital anatomy would allow females to regain some control over copulation and/or fertilization success [12]. These female morphological adaptations would select for additional adaptations in the male anatomy, resulting in coevolution of male and female structures [6], [13].

Birds have generally not been subject to studies of genitalia evolution because most male birds lack any external or complex genitalia. Only 3% of all avian species possess a phallus, or intromittent organ [14], and these species are all members of basal lineages of extant birds [14], [15]. The only avian group for which a comparative morphological study of male genitalia has been conducted is waterfowl (Aves: Anatidae) [16]. In male waterfowl the phallus is highly variable in both length (1.25–>40 cm) [16], [17], and the degree of elaboration (smooth, or covered with spines and grooves) [16], [18], and across species these variations are positively correlated with the frequency of forced extra-pair copulation (FEPCs) [16]. The avian phallus may allow males to achieve intromission without female cooperation [14], [18], and to deposit semen closer to the site of sperm storage and/or fertilization to increase their likelihood of fertilization [14], thereby providing males with a copulatory advantage over females.

In many taxa there is evidence that females respond to manipulating male strategies with behavioural counter-strategies to retain control over fertilization [reviewed in 12]. In several invertebrates, the female response to male reproductive strategies involves changes in genital anatomy [13], [19]–[22], although in general female genitalia are less variable than male genitalia [1]. The avian vagina has invariably been described as a short, narrow muscular duct, folded upon itself and covered with connective tissue [23] and no variation in this basic design has been reported. However, given the variability in the anatomy of the waterfowl phallus and its potential role in facilitating FEPCs, we hypothesized that female waterfowl would have evolved anatomical adaptations in response to the phallus to retain control over insemination and fertilization.

## Results

**Figure 1:**
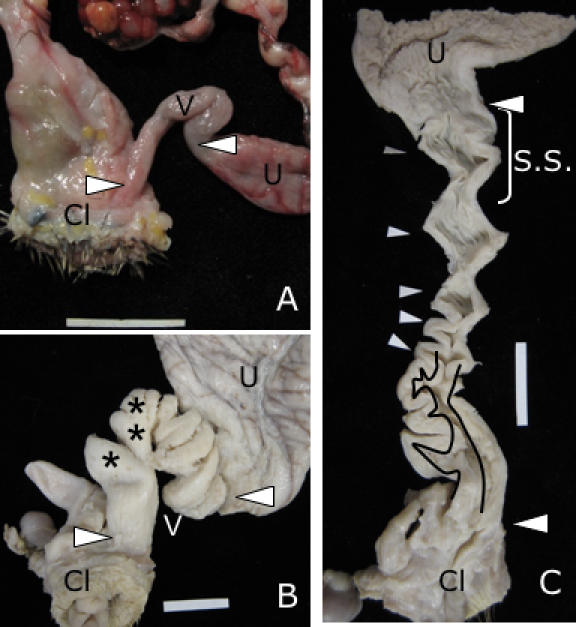
Avian vaginal morphology. (A) Typical tubular avian vagina from domestic Pheasant (*Phasianus colchicus*) (connective tissue removed). Note the lack of any elaborations. (B) Vagina (V) of Pekin duck (domestic *Anas plathyrhynchos*) (connective tissue removed). Note the complexity of the structure. (C) Longitudinal dissection of Pekin Duck vagina showing structural complexity. Pockets (*) are closer to the cloaca (Cl) and their lumen in shown between the traces lines. Spirals (white arrows) are closer to the uterus (or shell gland) (U). S.S. = Area of sperm storage tubules. (Scale bar in all pictures = 2 cm).

We examined vaginal and phallus anatomy in a sample of 16 waterfowl species, collected during the reproductive season. We found great variation among species in vaginal morphology. Some species had the typical simple avian vagina (Figure 1), whereas others had a highly complex vagina (Figure 1). Vaginal elaborations included a variable number of blind ending pouches proximal to the cloaca, and a variable number of clockwise spirals ending at the shell gland (or uterus) (Figure 1). Pouches are “dead end” side cavities in the vaginal lumen that cannot be eliminated by longitudinal elongation of the vagina. Pouches are located in the distal end of the vagina, close to the cloaca, and varied in number from 0–3 among species. Spirals are full 360° twists in the vagina that can be eliminated with elongation of the oviduct, and are found at the cranial end of the vagina always ending at the shell gland. Spirals varied in number from 0–8 among species. The magnitude of vaginal elaboration we found in waterfowl is surprising because no variations in vaginal morphology have been previously reported in birds despite decades of anatomical research on avian oviducts [23], [24].

Although the mechanics of copulation in birds with phalluses have not been studied, eversion of the male phallus occurs during, not prior to, cloacal contact (P. Brennan, pers. obs.). Thus, the shape and location of the vaginal pouches suggests that they might prevent the phallus from fully everting, and therefore from depositing sperm further inside the vagina. Our observations indicate that these pouches do not function in sperm storage: examination of the mucosal folds inside the vagina of Pekin duck (domestic *Anas plathyrhynchos*), Long-tailed duck (*Clangula hyemalis*), Widgeon (*A. americana*), Green-winged Teal (*A. carolinensis*) and African goose (*Anser cygnoides*) revealed sperm storage tubules (SSTs) only in the utero-vaginal junction, where they occur in all other avian species [25], and none inside the vaginal pouches (Figure 1). Sperm deposited in the vaginal pouches proximal to the cloaca would have a longer distance to travel to fertilize an ovum and may be more easily ejected by the female [26].

**Figure 2:**
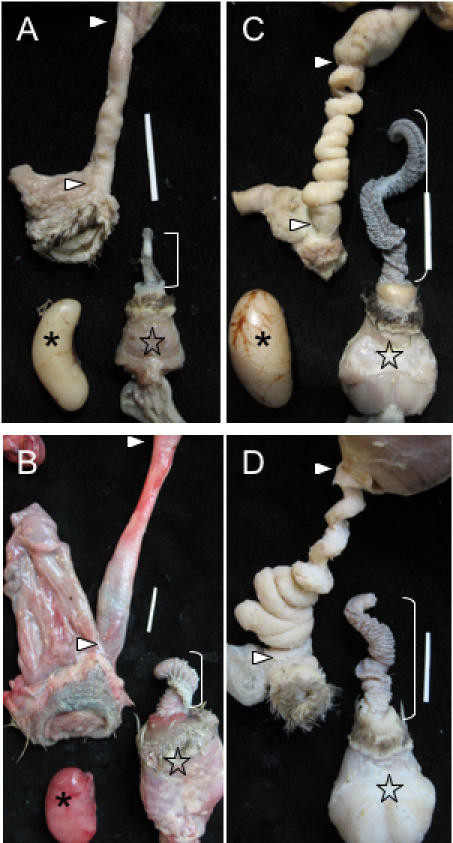
Examples of genital covariation in waterfowl. (A) Harlequin duck (*Histrionicus histrionicus)* and (B) African goose (*Anser cygnoides*), two species with a short phallus and no forced copulations, in which females have simple vaginas as in Fig 1a. (C) Long-tailed duck (*Clangula hyemalis)*, and (D) Mallard *Anas platyrhynchos* two species with a long phallus and high levels of forced copulations, in which females have very elaborate vaginas (size bars = 2 cm). ] = Phallus, * = Testis, ★ = Muscular base of the male phallus, ▹ = upper and lower limits of the vagina.

Congruent with previous descriptions [27], the phallus of all waterfowl species we examined spiralled in a counter-clockwise direction (viewed from the base of the phallus to the tip) (Figure 2), but the vaginal spirals we discovered were coiled in the opposite direction (moving from the cloaca to the shell gland)(Figure 2). Overall, the anatomy of these complex waterfowl vaginas suggests that pouches and spirals are anatomical barriers that function to exclude the male phallus. If this is the case, we would expect that male and female genital structures would have coevolved so that waterfowl species in which males have a longer phallus and higher levels of forced extra-pair copulations (FEPCs) would have a more elaborate vagina, while species where males have a small phallus and lower levels of FEPCs would have a simpler vagina.

**Figure 3:**
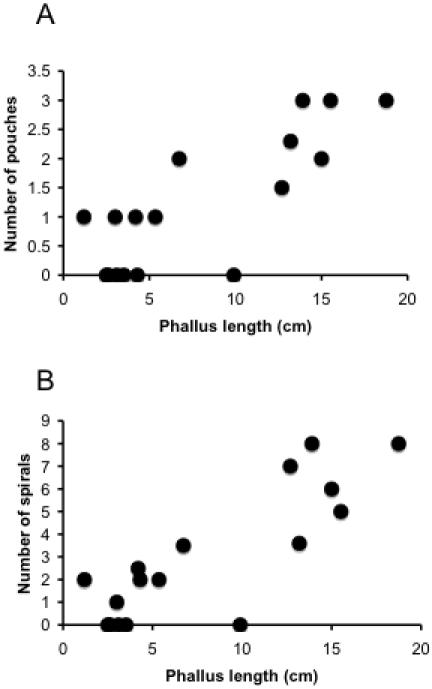
Relationships between male phallus length and female vagina. (A) Phallus length *vs.* number of vaginal pouches. (B) Phallus length *vs.* number of vaginal spirals. Points are the averages for each species studied.

To test this prediction we conducted a phylogenetically controlled comparative study of phallus size and vaginal morphology in 16 waterfowl species. We found great variation in the presence and number of both vaginal pouches and spirals among species. Consistent with our prediction, those species with a small phallus had short and simple vaginas, while species with a long phallus had longer and more elaborate vaginas (Figure 2). We performed comparative statistical analyses of the variation in phallus and vaginal morphology among species using a Generalized Least Squares method that controls for phylogenetic relationship (see Methods). Controlling for phylogenetic relationship, variation in phallus length was independent of male body mass (*b* = 0.11, *r* = 0.22, *P* = 0.35), and the number of vaginal pouches and spirals were independent of female body mass (pouches: *b* = 0.09, *r* = 0.19, *P* = 0.43; spirals: *b* = 0.04, *r* = 0.07, *P* = 0.77). The number of vaginal pouches and spirals were both significantly and positively correlated with male phallus length (pouches: *b* = 0.48, *r* = 0.55, *P* = 0.016; spirals: *b* = 0.84, *r* = 0.78, *P* = 0.00008, Figure 3) and with vaginal length (pouches: *b* = 1.14, *r* = 0.68, *P* = 0.0014; spirals: *b* = 1.37, *r* = 0.66, *P* = 0.002), suggesting that longer phalluses are associated with more elaborate vaginas and that longer vaginas are more elaborate.

Morphological elaborations of the waterfowl vagina could be functions of vagina length, however, vagina length itself has also been hypothesized to co-evolve as a response to increased phallus size and sperm length in several taxa [28]–[32]. Controlling for phylogeny, vagina length was correlated with both female body mass (*b* = 0.16, *r* = 0.63, *P* = 0.004) and phallus length (*b* = 0.39, *r* = 0.75, *P* = 0.0002). To explore the independent effects of vaginal length and phallus length on the number of vaginal spirals and pouches, we conducted a partial correlation analysis based on the correlation coefficients calculated from Continuous (see Methods). Even after removing the effect of vaginal length, the correlation between phallus length and number of spirals was still significant (*r* = 0.579, *DF* = 13, *t* = 2.56, *P* = 0.02), but not between phallus length and number of pouches (*r* = 0.07, *DF* = 13, *t* = 0.26, *P* = 0.8). Removing the effect of phallus length, however, resulted in non-significant correlations between vaginal length and both number of spirals (*r* = 0.187, *DF* = 13, t = 0.69, *P* = 0.5) and number of pouches (*r* = 0.494, *DF* = 13, *t* = 2.05, *P* = 0.06).

When vagina length is held constant, phallus length continues to explain variation in the number of spirals (but not pouches), whereas holding phallus length constant reveals that vaginal length alone does not explain either number of spirals or pouches. After controlling for the effect of female mass, vagina length is still significantly correlated with number of pouches (*r* = 0.746, *DF* = 13, *t* = 4.04, *P* = 0.001) and spirals (*r* = 0.721, *DF* = 13, *t* = 3.75, *P* = 0.002).

These results suggests that female vaginal elaborations are not the result of females simply having longer vaginas, but that vaginal morphology and length covary with male phallus length. For example, the longest vagina is found in one of the smallest ducks, *Oxyura dominica,* which also has one of the longest phalluses.

**Figure 4:**
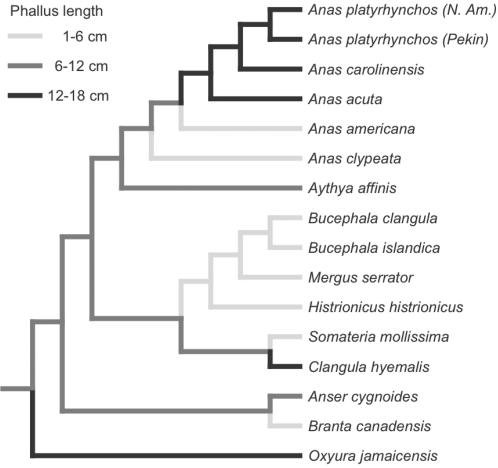
Hypothesis of the phylogenetic pattern of evolution in phallus length, based on the phylogeny proposed in Fig. S1, using the minimized squared change parsimony algorithm in MacClade, and three size classes (1–6 cm, 6–12 cm, and 12–18 cm). Phallus length >12 cm has evolved three times independently within these waterfowl- in *Oxyura, Clangula*, and *Anas.* All three of these lineages show correlated evolution of anatomical counter measures in the female reproductive tract.

A phylogenetic analysis of phallus length evolution in this sample of 16 waterfowl species indicated that large phallus size has evolved independently and convergently in at least three lineages: stiff-tailed ducks (*e.g.* *Oxyura*), dabbling ducks (*e.g.* *Anas*) and diving ducks (*e.g.* *Clangula*) (Figure 4). Phylogenetic analyses of the number of pouches and spirals, and vaginal length demonstrate that these correlated specializations evolved independently in all three lineages of waterfowl with large phallus size (e. g. *Anas* and *Clangula* shown in Fig. 2; see Table S1: Supporting materials). Although a more complete taxonomic sample will further resolve these macro-evolutionary patterns, it is clear that sexual covariation in waterfowl genital anatomy is not a simple monotonic trend, but a complex pattern that includes both correlated reduction and elaboration in different lineages.

## Discussion

The complex genitalia of female waterfowl are the first reported for any avian group. Elaborate vaginal morphology appears to have coevolved with male phallus length, which in turn covaries with levels of forced extra-pair copulation [16]. These data represent the most elaborate known case of genital coevolution in vertebrate animals.

Phallus length is positively correlated with both vagina length and the number of vaginal elaborations. Vagina length has been shown to coevolve with male genital traits (phallus length and sperm characteristics) in a number of taxa [28]–[32] as a result of intersexual selection. Therefore, it is likely that vagina length in waterfowl represents another correlated female morphological adaptation to the phallus. Vaginal length alone does not explain, and it is unlikely to be the direct cause of the vaginal elaborations in waterfowl, because there is great variation among other birds in vagina length without any of the morphological complexities we have described here. Partial correlation analysis showed that phallus length continues to explain variation in the number of spirals (but not pouches) when vagina length is held constant, whereas vaginal length alone does not explain either number of spirals or pouches when phallus length is held constant. Our results combined show that variation in phallus length, vagina length, and vaginal elaborations are all phylogenetically correlated, but that vagina length does not have significant individual correlation with vaginal elaboration.

The pattern of coevolution of genitalia in waterfowl we discovered could be explained by genital homology, natural selection, and different mechanisms of sexual selection.

### Homology

The observed correlation between male phallus length and female vaginal elaboration could occur if the traits were homologous. Selection acting on elaboration of a trait in one sex can lead to correlated elaboration of a homologous trait in the other sex [33]. However, the coevolved genital structures of male and female waterfowl are not homologous. The female oviduct originates from the Müllerian ducts [34], whereas the male phallus is derived from tissue from the ventral region of the cloaca and is homologous with the female hemi-phallus [27].

### Natural selection

The pouches and spirals in the female vagina could have evolved through natural selection alone. Since most waterfowl copulate in the water [35], vaginal spirals might prevent water from entering the reproductive tract during copulation if they form a tight seal at the entrance of the shell gland. If the risk of water entering the vagina is proportional to phallus length, this could explain why spirals are present only in species with longer phalluses. However, this hypothesis alone cannot explain either why the vaginal spirals twist in the opposite direction of the male phallus, or the presence of vaginal pouches. A critical test of this natural selection hypothesis would be whether waterfowl that copulate on land and have a long phallus lack the vaginal spirals. Few waterfowl copulate exclusively on land (e.g. Magpie Goose, *Anseranas semipalmata*, Hawaiian Goose *Branta sandvicensis,* and Cape Barren Goose, *Cereopsis novae-hollandiae* [35]) but no female specimens of any of these species were available for our study. Lastly, living crocodilians copulate submerged under water [e.g. 36], and male crocodilians have a phallus [27]. However, female crocodilian oviducts apparently lack any of the morphological elaborations observed in waterfowl [37].

Natural selection against hybridization (i.e. reinforcement) can lead to the evolution of a genital “lock and key” mechanism and complex genitalia [38]. This hypothesis predicts coevolution between male and female genitalia because male genitalia (the key) must match female genitalia (the lock), in order for successful copulation to take place [1]. However, since the female waterfowl vagina spirals in the opposite direction to that of the male's phallus, this suggests antagonistic rather than mutualistic coevolution that does not support the “lock and key” hypothesis.

### Sexual selection

Coevolution of male and female genital morphology has been hypothesized to result from intersexual selection via female choice for males that are good stimulators or of higher quality [1], [5] or from an arms race between the sexes over the control of insemination and fertilization [1], [2], [6]–[8]. Distinguishing between these mechanisms is not possible with our morphological results alone. However, the suggested role of male manipulation via the phallus [14], [18] and female resistance during FEPCs [39], [40], suggests that intersexual selection is likely responsible for the observed coevolution of genitalia in waterfowl. The female morphology we discovered strongly suggests that vaginal genital novelties function as a barrier to phallus penetration, and FEPCs might be responsible for their evolution in waterfowl. Although it had been previously suggested that the anatomical and physiological characteristics of the avian cloaca should allow the females to manage semen, and reduce the likelihood of successful forced copulations [41], the results presented here provide the first evidence of a macro-anatomical adaptation in the female oviduct that can also potentially serve as a mechanism of cryptic female choice. It is possible that the very low fertilization success of FEPCs in those waterfowl species for which genetic data exist [42]–[46] reflect the female's ability to retain control of fertilization.

A previous comparative study of male phallus anatomy in waterfowl concluded that phallus size and structural elaboration have evolved through sperm competition. The authors assumed that the spines and ridges found in the phallus of some waterfowl function to remove rival sperm from the female's vagina in the species at higher risk of sperm competition (those that engage in more forced copulations) [16]. Our study of female anatomy strongly suggests that intersexual selection is an additional, and perhaps the primary, selective force in the evolution of diversity in size and elaboration seen in male waterfowl genitalia.

## Materials and Methods

### Specimens

We collected oviducts, phalluses and testes from males and females of 16 waterfowl species (Table S1: Supporting materials). Specimens of 13 species were collected during the reproductive season, all of which were socially paired at the time of collection. Two species were obtained from commercial farms (Pekin Duck from the UK and African Geese from the USA). Reproductive organs from *Mergus serrator* were collected from specimens in breeding condition deposited in the University of Alaska Fairbanks Museum. Birds were weighed and dissected in the field as soon as they were collected, or frozen the day of collection and dissected in the laboratory. Only measurements from males and females with well developed gonads were included in the analysis, because avian reproductive organs regress outside of the breeding season [23]. The phallus was manually everted until the entire phallus was exposed and the ostium (or distal tip) had been reached. Length measurements were taken from fully everted, formalin-fixed phalluses by using dental floss placed inside the sulcus of the phallus from the base to the tip. The testes were collected, weighed, and used to determine reproductive status of the male. Sexual status of the female was determined upon evaluation of the ovary, as indicated by the presence of eggs in the oviduct or a well developed oviduct. Female oviducts and ovaries were preserved in formalin 10%, and all connective tissue around the vagina was removed to expose the underlying shape. The length of the vagina was measured as the distance between the rim of the cloaca and the uterovaginal junction after stretching and dissecting the vagina longitudinally and following the inside length of a single vaginal fold switching to the nearest fold if the original fold disappeared.

### Molecular phylogeny

A phylogenetic hypothesis for 18 waterfowl species (including two outgroup species from the basal waterfowl genus *Dendrocygna*) was estimated using Bayesian analysis of DNA sequence data, as implemented in MrBayes [47] and was used for all the phylogenetic analyses reported here. A combined data set comprising three mitochondrial (mtDNA) genes (cyt-b, ND2, 12S) and portions of four nuclear genes (CD4, LCAT, PEPCK, alpha hemoglobin) was analyzed using a mixed-model framework in which separate base composition and substitution matrix parameters were estimated for each data partition (Figure S1: Supporting materials). Both North American and Eurasian mitochondrial lineages of *Anas platyrhynchos* were included to reflect the close relationship of Mallard and its domesticated descendant, Pekin Duck. Mitochondrial data for *Anser cygnoides* were not available so we substituted sequences from a congener, *Anser albifrons*. Phylogenetic data used *Merganser merganser,* which is the sister species to *Merganser serrator* used in the morphological observations. All but three branches had posterior probabilities of 100% and two of the remaining branches were >95%; only the relationships among *Anas acuta*, *A. platyrhynchos,* and *A. carolinensis* were uncertain, the latter two species forming a clade in 52% of sampled trees.

### Phylogenetic Analysis

Comparative statistical analysis was performed using Continuous 1.0d13 [48], [49], which applies a generalized least squares (GLS) model to account for the shared phylogenetic history —phylogenetic covariance—between trait values of different species based on a matrix of shared evolutionary distances among species. Using the topology and branch lengths from the phylogeny, we examined the correlation between mass and genital morphology, and male and female genital morphology from all specimens in reproductive condition. Values of all variables were log transformed. Before log transformation, 1 was added to each value for the female morphology variables–the number of pockets and the number of spirals–to eliminate zero values. A constant-variance (random walk) GLS model was indistinguishable from the directional change model (≠0) using the Log Ratio Test (P = 0.99). All analyses were then conducted under constant variance assumptions, with all other scaling parameters equal to 1, the default values. The significance of each correlation was tested separately with a Log Ratio test comparing the nested hypotheses of no correlation (null) vs. correlation. Correlation coefficients were calculated by dividing the character covariance of the independent and dependent variables by the variation of the independent variable [48], [49]. Partial correlation analysis was conducted using these correlation coefficients and using the standard formulae [50]. Phylogenetic patterns in the continuous character variation were analyzed using MacClade [51] with the least squared parsimony logarithm.

## Supporting Information

Table S1 Length (cm) and elaboration of waterfowl genitalia.
(0.06 MB DOC)

Figure S1 Molecular phylogeny of waterfowl species used in the comparative analysis. Bayesian posterior probabilities (PP) are indicated in red for those nodes with less than 100% PP.
(0.09 MB DOC)
