# Supplementary material for: Coevolution of Male and Female Genital Morphology in Waterfowl
Source: PLoS One. 2007 May 2;2(5):e418. doi: 10.1371/journal.pone.0000418 (PMC1855079; doi:10.1371/journal.pone.0000418)
Supplement: Table S1 — Length (cm) and elaboration of waterfowl genitalia. (0.06 MB DOC) [file pone.0000418.s001.doc]

Table S1. Length (cm) and elaboration of waterfowl genitalia.

| Species | Sex | N | Phallus length | Sex | N | Vaginal length | Pouches | Spirals |
| --- | --- | --- | --- | --- | --- | --- | --- | --- |
| *Anas acuta* | M | 1 | 18.7 | F | 2 | 17 | 3 | 8 |
| *Anas americana* | M | 5 | 5.3 | F | 1 | 8.5 | 1 | 2 |
| *Anas clypeata* | M | 2 | 4.2 | F | 2 | 7.82 | 1 | 2.5 |
| *Anas carolinensis* | M | 6 | 13.9 | F | 3 | 12.6 | 3 | 8 |
| *Anas platyrhynchos* | M | 2 | 13.2 | F | 3 | 13.7 | 2 | 3.6 |
| *Aythya affinis* | M | 4 | 6.7 | F | 2 | 10.2 | 2 | 3.5 |
| *Branta canadensis* | M | 1 | 2.5 | F | 1 | 10.7 | 0 | 0 |
| *Bucephala clangula* | M | 1 | 3.1 | F | 1 | 6.4 | 0 | 0 |
| *Bucephala islandica* | M | 1 | 3 | F | 1 | 7.2 | 1 | 1 |
| *Clangula hyemalis* | M | 1 | 12.7 | F | 2 | 11 | 1.5 | 7 |
| *Histrionicus histrionicus* | M | 1 | 3.5 | F | 1 | 6.7 | 0 | 0 |
| *Mergus serratus* | M | 2 | 4.3 | F | 1 | 8.12 | 0 | 2 |
| *Oxyura jamaicensis* | M | 1 | 15 | F | 1 | 18 | 2 | 6 |
| *Somateria mollissima* | M | 2 | 2.6 | F | 1 | 8.63 | 0 | 0 |
| *Anas domesticus** | M | 3 | 15.5 | F | 2 | 17.2 | 3 | 5 |
| *Anser cygnoides** | M | 1 | 9.9 | F | 1 | 12.5 | 0 | 0 |

*Specimens from commercial farms
