# Supplementary material for: Coevolution of Male and Female Genital Morphology in Waterfowl
Source: PLoS One. 2007 May 2;2(5):e418. doi: 10.1371/journal.pone.0000418 (PMC1855079; doi:10.1371/journal.pone.0000418)
Supplement: Figure S1 — Molecular phylogeny of waterfowl species used in the comparative analysis. Bayesian posterior probabilities (PP) are indicated in red for those nodes with less than 100% PP. (0.09 MB DOC) [file pone.0000418.s002.doc]

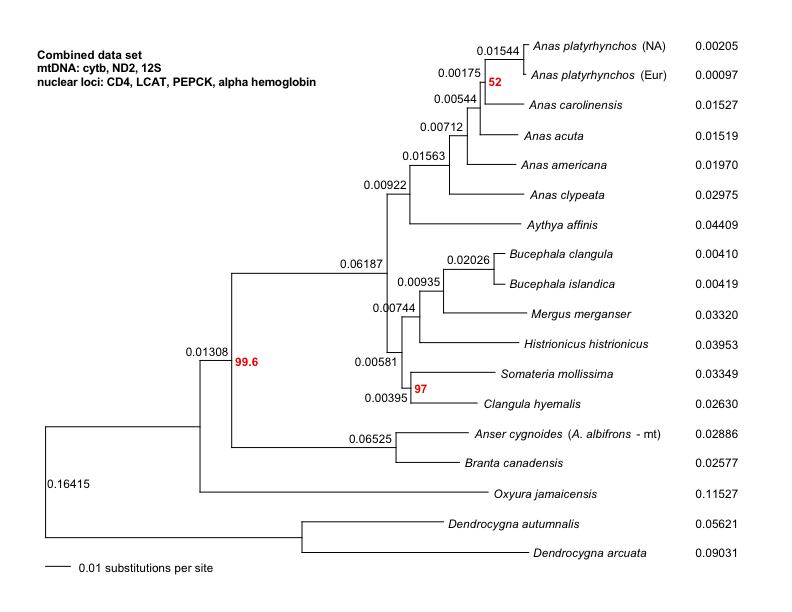


**Figure S1. Molecular phylogeny of waterfowl species used in the comparative analysis. Bayesian posterior probabilities (PP) are indicated in red for those nodes with less than 100% PP.**
